# A Wnt-planar polarity pathway instructs neurite branching by restricting F-actin assembly through endosomal signaling

**DOI:** 10.1371/journal.pgen.1006720

**Published:** 2017-04-06

**Authors:** Chun-Hao Chen, Chun-Wei He, Chien-Po Liao, Chun-Liang Pan

**Affiliations:** Institute of Molecular Medicine, College of Medicine, National Taiwan University, Taipei, Taiwan; The University of North Carolina at Chapel Hill, UNITED STATES

## Abstract

Spatial arrangement of neurite branching is instructed by both attractive and repulsive cues. Here we show that in *C*. *elegans*, the Wnt family of secreted glycoproteins specify neurite branching sites in the PLM mechanosensory neurons. Wnts function through MIG-1/Frizzled and the planar cell polarity protein (PCP) VANG-1/Strabismus/Vangl2 to restrict the formation of F-actin patches, which mark branching sites in nascent neurites. We find that VANG-1 promotes Wnt signaling by facilitating Frizzled endocytosis and genetically acts in a common pathway with *arr-1*/β-arrestin, whose mutation results in defective PLM branching and F-actin patterns similar to those in the *Wnt*, *mig-1* or *vang-1* mutants. On the other hand, the UNC-6/Netrin pathway intersects orthogonally with Wnt-PCP signaling to guide PLM branch growth along the dorsal-ventral axis. Our study provides insights for how attractive and repulsive signals coordinate to sculpt neurite branching patterns, which are critical for circuit connectivity.

## Author summary

Extrinsic cues instruct neurite branching patterns through cytoskeletal remodeling at precise locations. We show that the Wnt glycoproteins signal through the Frizzled receptor and the Planar Cell Polarity (PCP) protein VANG-1 to instruct neurite branching in the nematode *C*. *elegans*, by restricting F-actin assembly to positions along the nascent neurite where future branches emerge. VANG-1 facilitates Frizzled endocytosis and its subsequent signaling in the endosome, which is critical for shaping F-actin assembly. This Wnt-PCP-endosomal pathway intersects orthogonally with Netrin signaling to pattern neurite branching in the two-dimensional space. Our study uncovers a novel role for repulsive Wnt signaling in patterning neurite branches and illustrates how instructive cues along orthogonal body axes coordinate to define neural circuit connectivity.

## Introduction

Branching of the axon or dendrite expands the connectivity of neural circuits and is critical for the functions of the nervous system. Transcription factors have been shown to specify the morphology of neuronal branch arbors as part of cell fate determination [1]. In addition, diffusible, secreted cues also regulate neurite branching. For example, locally applied nerve growth factor (NGF) or Netrin-1 induce *de novo* interstitial branch formation in cultured cortical neurons [2, 3]. Neurite branching is also patterned by inhibitory signals. In the amphibian and vertebrate visual systems, repulsive ephrin-Eph signaling shapes topographic innervation of tectal neurons by preventing ectopic branching of retinal ganglion cells (RGC) beyond the target zones [4–6]. Furthermore, graded Wnt glycoproteins repel the chick RGC axon branches in the tectum [7]. These studies highlight the importance of inhibitory cues in instructing neurite branching patterns.

How extracellular signals remodel neuronal cytoskeleton to generate branches at specific locations is incompletely understood. Previous studies suggest that focal enrichment of filamentous actin (F-actin) is an early molecular signature for axon branch formation, which precedes the development of protrusive membrane activity and subsequent branch outgrowth [8–11]. Adhesion receptors instruct axon branches of the hermaphrodite-specific neuron (HSN) in *C*. *elegans* by locally promoting F-actin assembly [12]. A recent study in *Drosophila* suggests that inter-neuronal interaction of transmembrane protein Dscam1 specifies dendrite branching sites by regulating F-actin dynamics through kinases such as DOCK and Pak [13]. These studies provide a link between attractive cues and F-actin assembly in defining axon branching sites [14]. It is less clear how the repulsive signals engage neuronal cytoskeleton to pattern neurite branching.

In the present study, we uncover a role for secreted Wnt glycoproteins in specifying the stereotyped branching pattern of the PLM mechanosensory neurons in *C*. *elegans*. In the mutants of *Wnts*, *Frizzled* receptors or the planar cell polarity (PCP) gene *vang-1/Strabismus/Vangl2*, the PLM branch develops at ectopic sites preceded by aberrant F-actin distribution, suggesting that Wnt-Frizzled/PCP signaling spatially patterns F-actin assembly to instruct branching sites. Our results suggest that VANG-1 promotes Wnt signaling by facilitating Frizzled endocytosis, and that endosomal localization of Frizzled is crucial for patterning PLM branching.

## Results

### Focal enrichment of filamentous actin precedes *C*. *elegans* neurite branching

**Fig 1:**
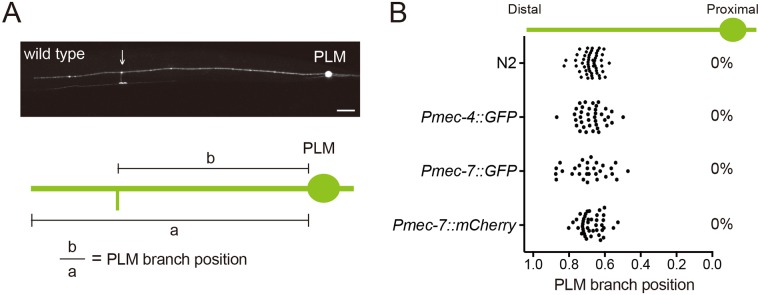
The PLM branching positions are highly predictable. (A) Schematic diagram of PLM morphology. (B) PLM branching pattern identified by immunostaining of N2 wild type with K40-acetylated tubulin antibody or by the transgenes *zdIs5(Pmec-4*::*GFP)*, *jsIs973(Pmec-7*::*RFP)* and *muIs42(Pmec-7*::*GFP)*. The percentage of mislocalized PLM branching sites was shown at the right of the distribution plot. N > 25.

The PLMs are bilaterally symmetric touch mechanosensory neurons in *C*. *elegans*, with a single collateral branch extending from the long anterior process that forms chemical synapses with interneurons in the ventral nerve cord (Fig 1A). The development of the PLM branch begins at late embryonic stages and is complete by 12 hours post-hatching in the wild-type animals [15]. The PLM branching sites were remarkably predictable in wild-type animals at the fourth larval (L4) stage when visualized with PLM-specific transgenes or by staining the animals with an antibody for acetylated microtubules (Fig 1B). This pattern was preserved throughout larval development (S1A Fig). We observed one and only one branch for each neuron at all time points, implying that PLM branching pattern is not shaped by pruning of unwanted branches (S1B Fig), as recently reported in the mammalian cortex [16].

**Fig 2:**
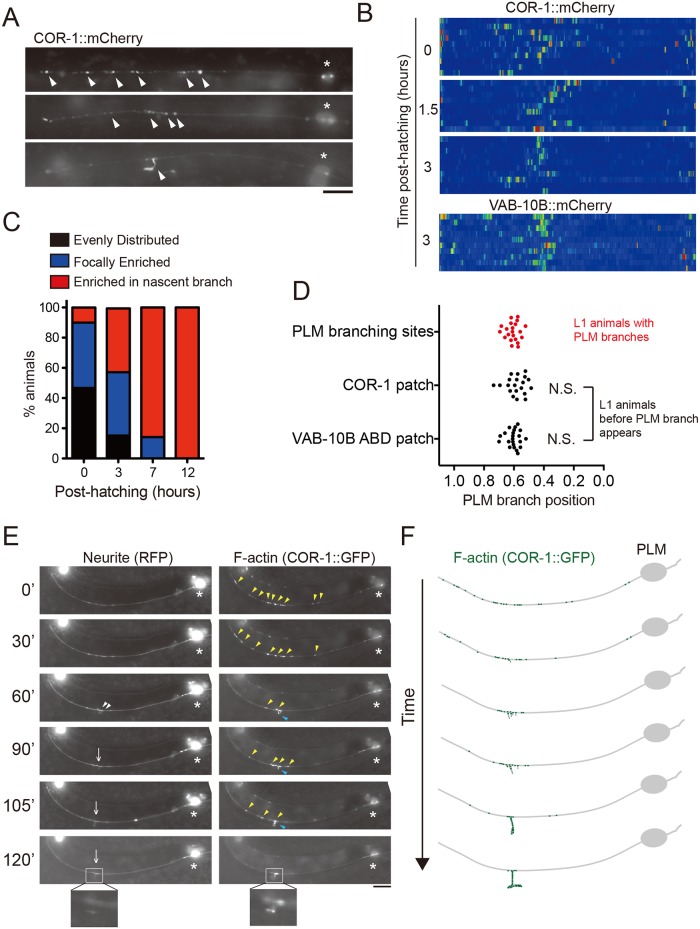
F-actin assembly precedes the future PLM branching sites. (A) Representative epifluorescent images of COR-1::mCherry distribution (arrowheads) in the PLM process of animals expressing *twnEx195(Pmec-7*::*COR-1*::*mCherry)*. Asterisks mark the PLM soma. Scale bars = 20 μm. (B) Heat maps of COR-1::mCherry and VAB-10B::mCherry*(twnEx256)* intensity in the PLM process. Synchronized animals were analyzed at indicated developmental stages. N = 10 animals each. (C) F-actin distribution in the PLM process at different developmental stages shows transition from random distribution to focal enrichment. N > 20. (D) Distribution of F-actin signal labeled by the F-actin markers, COR-1::mCherry and VAB-10B::mCherry, and PLM branching sites at L1 larval stage. N.S., not significant; F-test. N > 25. (E) Dual color fluorescent time-lapse imaging and (F) schematic diagram of PLM neuron and COR-1 in early L1 larvae. 14 early L1 larvae were analyzed and showed similar progressive enrichment of F-actin signal. Of these, seven animals developed a normal-appearing PLM branch. Others developed the branch but arrested before a synaptic varicosity formed, likely due to phototoxicity or desiccation. Time after hatching (minutes) was indicated. White arrowheads, filopodia; yellow arrowheads, F-actin signals; blue arrowheads, F-actin in the nascent branch; arrows, PLM branch. Scale bars = 10 μm.

We set out to determine how branch outgrowth is initiated at well-defined locations along the anterior-posterior (A-P) body axis. In search for molecular signatures that predict future branching sites in the unbranched PLM process, we expressed the F-actin binding protein COR-1/Coronin-1 or the actin binding domain of VAB-10B fused with fluorescent mCherry to label F-actin. We found that F-actin transitioned from a diffuse distribution along the PLM process to a more restricted localization in the distal region (Fig 2A–2C). Importantly, the locations of the F-actin patches in nascent, unbranched neurites strongly correlated with the PLM branching sites at L1 stage (Fig 2D), suggesting that focal enrichment of F-actin in nascent neurites defines future branching sites.

To test this, we performed time-lapse imaging to trace F-actin distribution during PLM branch outgrowth (Fig 2E and 2F). We observed that initially COR-1::GFP puncta were broadly distributed along the nascent, unbranched PLM neurite, followed by progressively restricted localization that eventually formed a bright F-actin patch (Fig 2E). Robust local protrusive activity of the neurite membrane was initiated after formation of the F-actin patch, and was subsequently stabilized with one collateral branch extending among several slender filopodia (Fig 2E and 2F). This nascent branch later reached the ventral midline and formed a presynaptic varicosity that connected with the ventral nerve cord. Of seven animals that we successfully recorded and generated a mature PLM branch tipped with a presynaptic varicosity, the PLM branch always developed from the brightest F-actin patch. This observation strongly indicates that the focal F-actin patch instructs PLM branch outgrowth.

### Wnt signals control PLM branching patterns by restricting F-actin assembly to future branching sites

**Fig 3:**
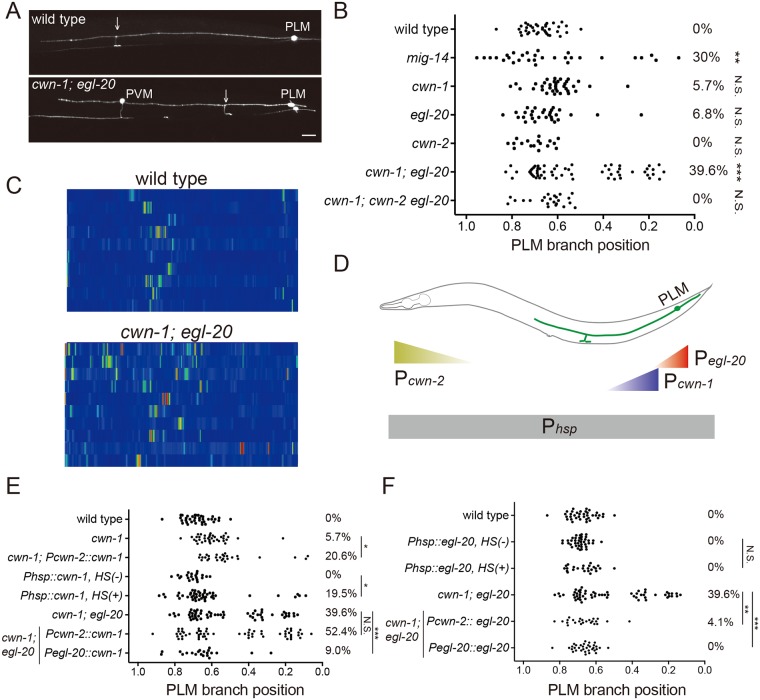
The Wnt glycoproteins patterns PLM branching positions. (A) Confocal images of PLM branches in the wild type and *cwn-1; egl-20* mutant animals. Arrows mark the PLM branch. PVM is another touch neuron. Scale bar = 10 μm. (B) Quantification of PLM branching positions in the *Wnt* mutants. Statistics was comparison between the wild type and each individual mutant strains. **, *p* < 0.01; ***, *p* < 0.005; N.S., not significant, two proportion z test with Bonferroni correction. N > 25. (C) Heat maps of COR-1::mCherry*(twnEx195)* intensity in the PLM process of wild-type and *cwn-1; egl-20* mutants three hours post-hatching. N = 10 animals each. (D) Diagram of expression patterns of the *hsp-16*.*2*, *cwn-2* or *egl-20* promoters in *C*. *elegans*. (E, F) PLM branching pattern in Wnt rescue and mis-expression lines. HS, heat shock treatment. **, *p* < 0.01; ***, *p* < 0.005; N.S., not significant, two proportion z test with Bonferroni correction. N > 25.

With these observations, we hypothesize that extracellular signals instruct placement of the PLM branch by regulating the pattern of F-actin assembly in the PLM neurite. Wnt signaling had been shown to be important in cell fate determination, axon guidance and synapse formation. In *C*. *elegans*, Wnts functions as repulsive cues for neuronal migration, neurite extension and synapse formation [17–19]. To test whether Wnts pattern PLM branches, we first examined animals with a hypomorphic mutation in *mig-14/Wntless*, which encodes a transmembrane protein essential for Wnt secretion [20, 21]. This *mig-14* mutation significantly depleted extracellular Wnts and caused defective PLM morphology in around 30% of the animals [22, 23]. For the remaining PLM neurons with normal morphology, branching sites became random along the PLM process. By contrast, the length of the PLM neurites with ectopic branching was indistinguishable from that of the wild-type PLM (Fig 3A and 3B and S2A Fig). This result suggests that normal Wnt trafficking is required for PLM branching pattern. We next examined PLM branching patterns in individual Wnt mutants (*cwn-1*, *egl-20*, *cwn-2*, *mom-2*), except for *lin-44*, the mutation of which resulted in severe PLM polarity defects [17]. While the *cwn-1* and the *egl-20* single mutants had no overtly defects in PLM branching patterns, distribution of branching sites in the *cwn-1; egl-20* double mutant expanded significantly, especially proximal to the cell body, indicating that *cwn-1* and *egl-20* together control PLM branching sites (Fig 3A and 3B). A mutation in the Wnt *cwn-2* did not alter PLM branching pattern. However, the *cwn-2* mutation suppressed the proximal branching phenotype of the *cwn-1; egl-20* double mutant (Fig 3B), suggesting that *cwn-2* prevents distal branching. Taken together, these data indicate that combinatorial effects of spatially distinct Wnts ensure that the PLM branch forms at a fairly predictable anterior-posterior position.

In around 5% of *cwn-1; egl-20* animals, we observed two PLM branches distant to each other in the same neuron during L1 stage (S2B Fig). This phenotype was transient, as none of the *cwn-1; egl-20* animals had multiple PLM branches when examined at L4, suggesting that unknown mechanisms exist to select one from these two PLM branches and eliminate the other.

We found that, in contrast to the single, highly localized F-actin patch in the wild-type unbranched PLM neurite 3 hours post-hatching, F-actin became dispersed and showed increased intensity in the *cwn-1; egl-20* mutants (Fig 3C), which persisted as late as 7 hours post-hatching (S2C Fig). The high F-actin activity may explain in part the transient multiple-branch phenotype in the *cwn-1; egl-20* mutant. Overall, these observations suggest that Wnt signals restrict F-actin distribution to mark the future branching sites.

### EGL-20 acts permissively and CWN-1 functions instructively for PLM branch placement

To test whether Wnts instruct PLM branching sites, we first induced widespread CWN-1 and EGL-20 expression by heat shock. Widespread CWN-1, but not EGL-20, expression shifted PLM branches both proximally and distally (Fig 3D–3F). This result suggests that CWN-1 functions instructively and EGL-20 does not. Indeed, anterior EGL-20 expression from the *cwn-2* promoter rescued PLM branching pattern of the *cwn-1; egl-20* double mutant to the same level as done by EGL-20 expression from the endogenous *egl-20* promoter expressed in the posterior (Fig 3F). By contrast, while posterior expression of CWN-1 from the *egl-20* promoter mimicked the endogenous CWN-1 gradient and rescued defective branching patterns of the *cwn-1; egl-20* mutant, anterior CWN-1 expression failed to rescue (Fig 3E). Moreover, the mild proximal branching phenotypes of the *cwn-1* mutant became more severe when CWN-1 was expressed in the anterior (Fig 3E). These data indicate that *cwn-1* is a repulsive cue for PLM branch placement.

### The Frizzled receptor MIG-1 and the planar cell polarity protein VANG-1 control PLM branching pattern

**Fig 4:**
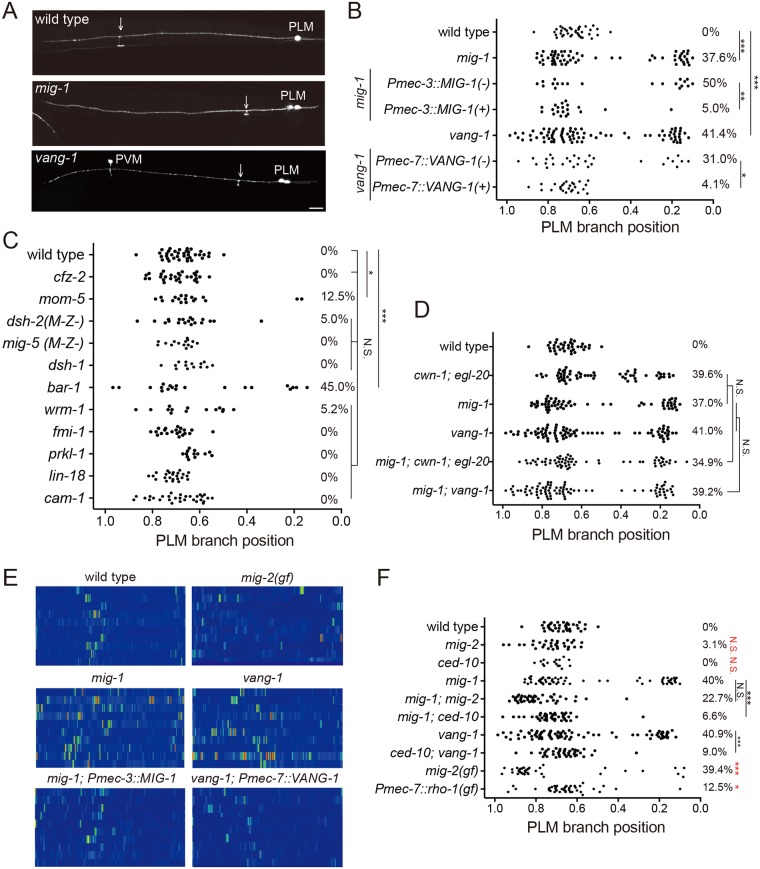
Frizzled and PCP components pattern PLM branching positions. (A) Confocal images of PLM branches in the wild type, *mig-1* and *vang-1* mutants. Arrows mark the PLM branch. PVM is another touch neuron. Scale bar = 10 μm. (B-D) PLM branching pattern in canonical and non-canonical Wnt pathway mutants. *, *p* < 0.05; **, *p* < 0.01; ***, *p* < 0.005; N.S., two proportion z test with Bonferroni correction. N > 25. (E) Heat maps of COR-1::mCherry*(twnEx195)* intensity in the PLM process of indicated genotypes three hours post-hatching. N = 10 animals each. (F) PLM branching pattern in indicated genotypes. **, *p* < 0.01; ***, *p* < 0.005; N.S., not significance, two proportion *z* test with Bonferroni correction. N > 25. Statistic results between wild-type and gain-of-function or loss-of-function *mig-2* and *ced-10* single mutants were shown in red. N > 25.

In our screen of the Wnt pathway mutants for defective PLM branching patterns, we found that mutations in the Frizzled receptor *mig-1*, the PCP transmembrane protein *vang-1* and the β-catenin *bar-1* caused abnormal PLM branching patterns (Fig 4A–4C). While Wnts, the Frizzled receptor and the β-catenin are all required for PLM branching patterns, we only observed mild phenotypes in *dsh-2*, one of the three Dishevelled mutants, likely due to functional redundancy between these genes. We focused on *mig-1* and *vang-1* because they showed comparable penetrance of PLM branching abnormality to that in the *cwn-1; egl-20* mutant. The proximal branching phenotype was not further enhanced in the *mig-1; cwn-1; egl-20* triple mutant compared to that of the *mig-1* or the *cwn-1; egl-20* mutant (Fig 4D), indicating that *mig-1* acts in a common pathway with *cwn-1* and *egl-20* and is likely the receptor for these two Wnts. Likewise, defects in PLM branching sites were not enhanced in the *mig-1; vang-1* double mutant compared to either single mutants, suggesting that *mig-1* and *vang-1* also function in the same pathway (Fig 4D). Similar to that in the *cwn-1; egl-20* double mutants, F-actin in the unbranched PLM neurite was increased and became dispersed in the *mig-1* and the *vang-1* mutants (Fig 4E). We also observed transient multiple-branch phenotypes in around 10% of *mig-1* or *vang-1* mutant animals, consistent with high, ectopic F-actin activity promoting branch outgrowth. Touch neurons-specific expression of *mig-1* or *vang-1* rescued the PLM branching defects as well as the aberrant F-actin patterns (Fig 4E), confirming that they act cell-autonomously in the PLM to pattern F-actin assembly and neurite branching.

### Wnt signaling restricts F-actin assembly and branching patterns by inhibiting Rac and Rho

Increased F-actin activity in the *mig-1*, *vang-1* or *cwn-1; egl-20* double mutants prompted us to examine the Rho and Rac small GTPases, which are important F-actin regulators and had been implicated in Wnt-PCP signaling [24]. We found that mutations in the Rac small GTPases *mig-2* and *ced-10* significantly suppressed defective branching patterns of the *mig-1* and *vang-1* mutants (Fig 4F). Importantly, the single *mig-2* and *ced-10* mutants displayed normal PLM branching patterns (Fig 4F). Moreover, the *mig-2(gm103)* gain-of-function mutant displayed proximal branching phenotypes as well as aberrant F-actin activity reminiscent of those in the *Wnt*, *mig-1* or *vang-1* mutant (Fig 4E). Expression of constitutively active RHO-1 in the touch neurons also caused defective PLM branching patterns (Fig 4F). These results suggest that the repulsive activity of Wnts in F-actin assembly and subsequent neurite branching in part acts through inhibition of Rac and Rho small GTPases.

### Endocytosis of MIG-1 is essential for the PLM branch placement and requires VANG-1

**Fig 5:**
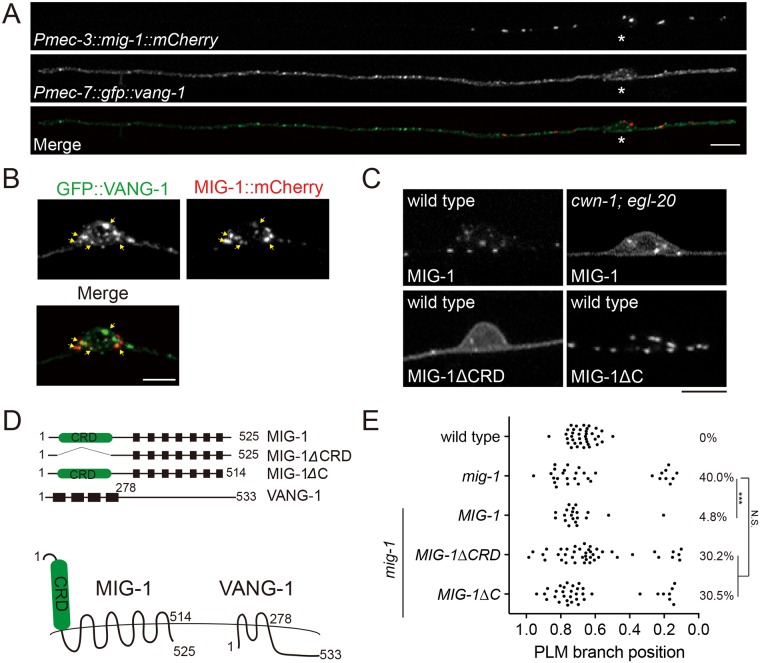
The cystein-rich domain and the C-terminal domain of MIG-1 are essential for PLM branching pattern. (A) Confocal images of MIG-1::mCherry and GFP::VANG-1 in the PLM. Asterisks, PLM soma. Scale bar = 10 μm. (B) Confocal images of MIG-1::mCherry and GFP::VANG-1 in the PLM cell soma. Arrows indicate colocalization of MIG-1 and VANG-1. Scale bar = 5 μm. (C) Distribution of wild-type or truncated MIG-1 in the PLM cell soma. **Δ**CRD, deletion of the cysteine-rich domain. **Δ**C, deletion of the C-terminus domain. Scale bar = 5 μm. (D) Schematic diagrams of MIG-1 and VANG-1 proteins. Black boxes are transmembrane domains. (E) PLM branching pattern. **, *p* < 0.01; N.S., not significance, two proportion *z* test with Bonferroni correction. N > 25.

By expressing MIG-1::GFP or MIG-1::mCherry chimeric proteins in the touch neurons, we found that MIG-1, mostly in punctate forms, was localized to both the plasma membrane and cytosol, as well as the proximal segment of the PLM neurite (Fig 5A and 5B). This highly polarized subcellular distribution of MIG-1 required Wnts, as in the *cwn-1; egl-20* double mutant, MIG-1 signal became diffuse over the plasma membrane and in the PLM process (Fig 5C). Clustering of MIG-1 on the membrane required the cysteine-rich domain (CRD) that binds Wnts, but not the short cytosolic tail (Fig 5C and 5D), while both were essential for transducing Wnt signaling to define the PLM branch patterns (Fig 5E).

As our genetic experiments suggest that EGL-20 acts permissively and CWN-1 functions as an instructive signal, we investigated whether EGL-20 and CWN-1 have distinct effects on the MIG-1 receptor. In the *egl-20* mutant, the punctate MIG-1 signals were markedly reduced, and MIG-1 became diffuse on the PLM membrane (S3A Fig). By contrast, punctate MIG-1 signals were not affected in the *cwn-1* mutant (S3A Fig). Expression of EGL-20 in the posterior fully restored the punctate MIG-1 signals, and anterior EGL-20 expression partially restored punctate MIG-1 distribution on the PLM membrane (S3A and S3B Fig). These data suggest that EGL-20 is important for clustering of MIG-1 on the plasma membrane, while CWN-1 may regulate MIG-1 in a different way.

**Fig 6:**
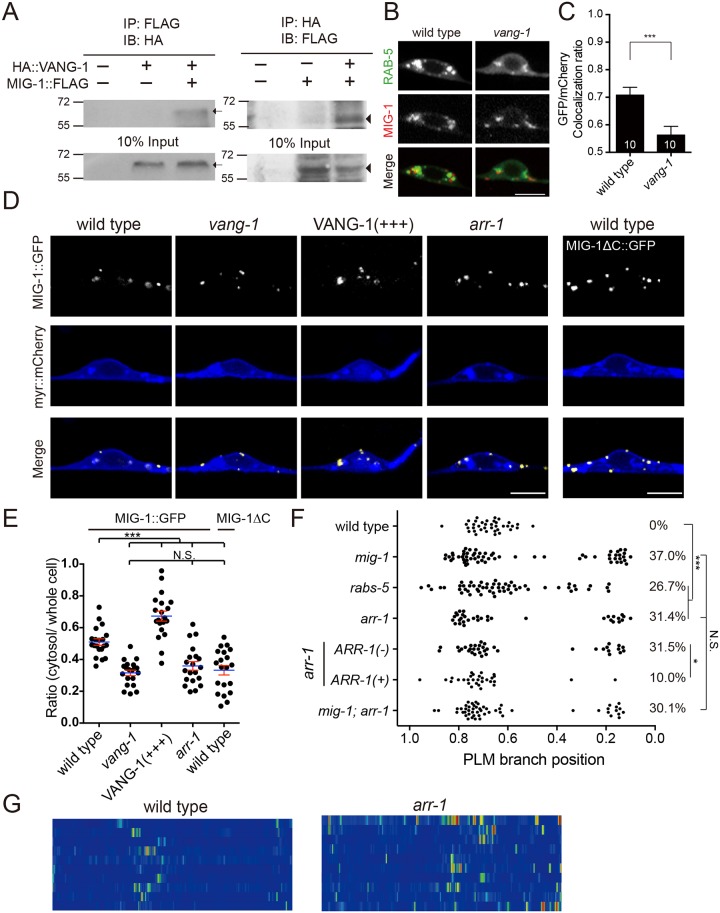
Frizzled signaling requires VANG-1-dependent endocytosis. (A) Co-immunoprecipitation of MIG-1 with VANG-1. Arrows and arrowheads indicate VANG-1 and MIG-1 bands, respectively. (B) Confocal images of MIG-1::mCherry and GFP::RAB-5 in the PLM soma. Scale bar = 5 μm. (C) Overlapping ratio of GFP (RAB-5) and mCherry (MIG-1) in the wild type and the *vang-1* mutant. Error bar = S.E.M. ***, *p* < 0.005, Mann-Whitney U test. N are numbers of scored cells. (D) Confocal images of MIG-1::GFP (pseudocolored in yellow) in the PLM soma. Myristoylated mCherry (myr::mCherry, pseudocolored in blue) labels the plasma membrane. Scale bar = 5 μm. (E) Quantification of MIG-1::GFP distribution in the PLM soma. The ratio of cytosolic/total MIG-1::GFP signal was quantified as described in *Materials and Methods*, under the section of *Confocal Microscopy and Quantification of MIG-1 and VANG-1 Subcellular Localization*. Each dot represents the MIG-1::GFP ratio for a single PLM neuron. Error bar = S.E.M. ***, *p* < 0.005; N.S., not significance, ANOVA. N > 20. (F) PLM branching pattern in *arr-1* and *rabs-5* mutants. *, *p* < 0.05; ***, *p* < 0.005; N.S., not significance, two proportion *z* test with Bonferroni correction. N > 25. (G) Heat maps of COR-1::mCherry*(twnEx195)* intensity in the PLM process of the wild type and the *arr-1* mutant three hours post-hatching. N = 10 animals each.

We found that GFP::VANG-1 was also in punctate forms but was evenly distributed along the PLM neurite, the cell membrane and in the cytosol (Fig 5B). When co-expressed, VANG-1 and MIG-1 puncta partially colocalized in the PLM neuron (Fig 5B). To test whether MIG-1 and VANG-1 form protein complexes, we ectopically expressed MIG-1 and VANG-1 in mammalian HEK293 cells and performed coimmunoprecipitation. We found that MIG-1 and VANG-1 coimmunoprecipitated each other, suggesting that they form protein complexes (Fig 6A). Of note, interaction with VANG-1 in the coimmunopreciptation experiments was independent of the CRD domain or the C-terminus of MIG-1 (S4 Fig).

A fraction of cytosolic MIG-1::GFP colocalized with the early endosome marker RAB-5, which was reduced in the *vang-1* mutant (Fig 6B and 6C). To further analyze MIG-1 distribution, we quantified MIG-1::GFP signals in series of single confocal optical sections through the thickness of the PLM soma, with myristoylated mCherry to label the plasma membrane (Fig 6D and 6E; see Materials and methods for detail of quantification). Elimination of the C-terminus domain of MIG-1 (MIG-1ΔC) resulted in its accumulation at the plasma membrane, confirming that the C-terminus is required for MIG-1 endocytosis (Fig 6D and 6E). MIG-1 localization to the plasma membrane was also increased in the *vang-1* mutant (Fig 6D and 6E). By contrast, VANG-1 overexpression resulted in increased MIG-1 cytosolic distribution and diminished membrane localization (Fig 6D and 6E), suggesting that VANG-1 controls MIG-1 endocytosis and subsequent localization in the early endosomes. We found that animals with a mutation in *arr-1/β-arrestin 2*, which is essential for Frizzled endocytosis [25], had defective branching and F-actin patterns similar to those in the *mig-1*, *vang-1* or *cwn-1; egl-20* double mutants (Fig 6F and 6G). Our genetic experiments further suggested that *arr-1* and *mig-1* acted in a common pathway and *arr-1* functioned in the PLM neuron (Fig 6F). MIG-1::GFP was more enriched on the plasma membrane in the *arr-1* mutant, similar to what was observed with MIG-1 that lacked the C-terminus tail (Fig 6D and 6E). In addition, mutations in RABS-5, a regulator of RAB-5-dependent endosomal trafficking, showed defective branching similar to that of the *mig-1* mutant, suggesting that endosomal trafficking (presumably of MIG-1) is required for proper PLM branching (Fig 6F). Based on these results, we propose that MIG-1 endocytosis is essential for transducing Wnt signals to pattern PLM branching locations, and that VANG-1 and ARR-1 control MIG-1 endocytosis.

### Orthogonal Wnt and Netrin cues pattern PLM branching along the anterior-posterior and dorsal-ventral body axes

**Fig 7:**
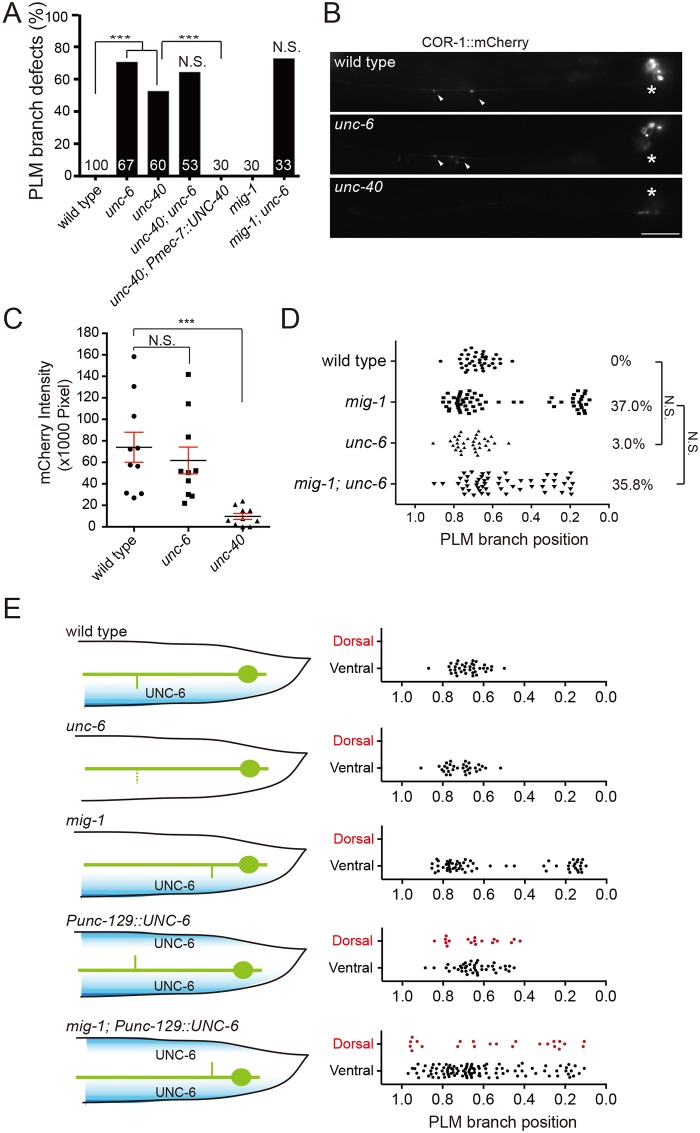
Netrin signaling controls dorsal-ventral growth of the PLM branch. (A) Quantification of branchless phenotypes in the Netrin pathway mutants. N are numbers of PLM neurons scored. ***, *p* < 0.005; N.S., not significant, two proportion z test. (B) Representative epifluorescence images of COR-1::mCherry(*twnEx195*) in the PLM process of the wild type and the mutants. Arrowheads, COR-1 puncta; asterisks, PLM cell bodies. Scale bar = 10 μm. (C) Quantification of COR-1::mCherry in the wild type, *unc-6* and *unc-40* mutants. Error bar = S.E.M. ***, *p* < 0.005, N.S., not significant, Mann-Whitney U test. (D) PLM branching pattern. N.S., not significant, two proportion *z* test. N > 30. (E) Diagram (left) and scoring (right) of PLM branching pattern. Blue shading indicates the ventral (normal) or dorsal (ectopic) UNC-6 gradient.

In the *mig-1*, *vang-1* and *cwn-1; egl-20* mutants, misplaced PLM branches still extend successfully to the ventral cord axons. Furthermore, we found that synaptic contacts were not disrupted in the *mig-1* mutant, judged by the GRASP(GFP Reconstitution Across Synaptic Partners) technique (S5 Fig) [26]. These observations suggest that mechanisms independent of Wnt signaling control PLM branch development along the dorsal-ventral (D-V) axis and later PLM synaptogenesis. Mutations in the axon guidance cue *unc-6/Netrin* and its receptor *unc-40/Deleted in Colorectal Cancer (DCC)* disrupted PLM branch growth along the dorsal-ventral (D-V) axis (Fig 7A). We found that F-actin activity in the nascent PLM neurite before branch outgrowth was significantly reduced in the *unc-40* but not the *unc-6* mutant (Fig 7B and 7C). This finding is consistent with the role of UNC-40 in regulating branch outgrowth by promoting F-actin assembly [27].

**Fig 8:**
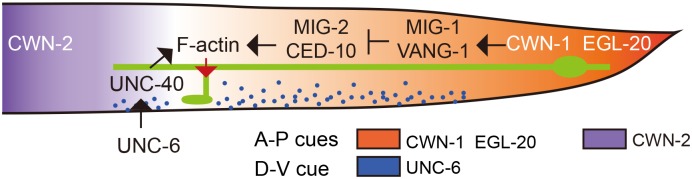
Model of PLM branch growth patterning by orthogonal Wnt and Netrin signaling. Schematic diagram depicting that Wnt and Netrin pathways orthogonally regulate PLM neurite branching. Secreted Wnt glycoproteins CWN-1, EGL-20 and CWN-2 are marked in orange and purple, respectively, which form discrete graded distribution along the anterior-posterior axis to regulate the stereotyped PLM branching pattern. It should be noted that, although shown to be distributed in a directional concentration gradient here, genetic experiments in this work suggest that EGL-20 functions somewhat independent of its expression patterns. UNC-6/Netrin acts through the DCC receptor UNC-40 to promote F-actin nucleation within a narrow zone along the anterior-posterior axis that is defined by the repulsive Wnt signaling at both ends. UNC-6, which is distributed in a concentration gradient that is high in the ventral, then promotes branch extension towards the ventral side.

While a significant percentage of the *unc-6* and *unc-40* animals lost their PLM branch, for those that developed branches, the branches were at wild-type locations along the PLM neurite (Fig 7D). This observation suggests that Wnts and ventrally-derived Netrin act orthogonally to pattern the PLM branch, with Wnts instructing the A-P position of the branch and Netrin promoting its growth along the D-V axis. To test this, we first analyzed *mig-1; unc-6* animals, and found that they showed comparable penetrance of missing and misplaced PLM branch to that in the *unc-6* and *mig-1* single mutants, respectively (Fig 7A and 7D). This suggests that *mig-1* and *unc-6* function independently. When *unc-6* was ectopically expressed in the dorsal musculature from a fragment of the *unc-129* promoter, the PLM branches grew at normal A-P locations but were sometimes misrouted dorsally (Fig 7E), suggesting that *unc-6* instructs D-V growth of the PLM branch. When *unc-6* was expressed dorsally in the *mig-1* mutant, we found that dorsally-routed PLM branches developed at ectopic locations along the A-P axis (Fig 7D). Together these experiments support the model that Wnts along the A-P axis and Netrin along the D-V axis interact orthogonally to pattern branch outgrowth in the PLM neuron (Fig 8).

## Discussion

In this study, we demonstrate that secreted Wnt glycoproteins, which show graded distribution along the A-P body axis [28], define neurite branching sites of the *C*. *elegans* PLM touch neuron. VANG-1- and arrestin-dependent Frizzled endocytosis transduces Wnt signals to polarize F-actin distribution, which precedes and correlates with collateral branch formation. In addition, Netrin promotes branch growth ventrally, orthogonal to the A-P positioning of the branch that depends on Wnts. Our work thus provides a conceptual framework for how extracellular signals intersect to instruct neurite branching in the 2D space.

### Inhibitory Wnts signals instruct neurite branching sites

Unlike the mammalian and avian nervous systems where pruning of excessive collateral branches shapes the final axon arbors [5, 16, 29], the *C*. *elegans* PLM neuron generates one and only one neurite branch at remarkably defined locations, suggesting that instructive mechanisms are involved. Previous studies indicate that Wnt signals function as inhibitory cues for neuronal growth cone migration and synapse formation [18, 19, 30, 31]. Our findings demonstrate that Wnt signals instruct PLM neurite branching by inhibiting F-actin assembly, expanding the roles of repulsive Wnt signaling in wiring the *C*. *elegans* nervous system connectivity. It would be interesting to test whether inhibition of synapse formation by Wnts also engages F-actin modulation, a mechanism recently shown to be critical for instructing synapse formation by adhesion receptors in the *C*. *elegans* hermaphrodite-specific neurons (HSN) [12].

### Permissive and instructive Wnt signals that pattern PLM branching sites

Functional redundancy among different Wnt molecules is well-established, but whether they signal in discrete ways at the molecular level is not adequately addressed. Our results suggest that CWN-1 is an instructive signal, while EGL-20 acts permissively to regulate PLM branching patterns. Permissive EGL-20 functions had been demonstrated in the polarization of the V5 seam cell or the posterior migration of the left Q neuroblast descendants [32, 33]. By contrast, EGL-20 functions as a repulsive signal in the migration of the hermaphrodite-specific neuron (HSN), projection of the AVM and PVM neurites and synapse formation in the DA motor neurons [19, 30]. Our genetic experiments imply that the Frizzled MIG-1 is likely a shared receptor for both CWN-1 and EGL-20. At the subcellular level, we found that EGL-20, but not CWN-1, clustered membrane MIG-1 receptors. In the absence of EGL-20, MIG-1 was delocalized and became diffuse on the membrane. Consistent with EGL-20 being a permissive signal for PLM branching pattern, clustering of MIG-1 could also be achieved in part by ectopic EGL-20 expression, although we cannot exclude the possibility that the anterior-expressing *Pcwn-2*::*EGL-20* transgene also expressed EGL-20 in the posterior at lower level. Clustering of MIG-1 *per se* does not seem to be essential for proper PLM branching, as ectopic PLM branching was minimal in the *egl-20* single mutant. Based on these observations, we speculate that EGL-20-dependent MIG-1 clustering facilitates activation of MIG-1 by the directional CWN-1 signal. In the *cwn-1* single mutant, other Wnts (such as MOM-2 and LIN-44) could compensate for the loss of CWN-1 and correctly pattern PLM branching. In the *egl-20* mutant, activation of MIG-1 by CWN-1 is less efficient but is still sufficient to correctly pattern PLM branching in most animals. When both CWN-1 and EGL-20 are removed, signaling through MIG-1 is largely abolished, resulting in significant ectopic branching. We reason that the use of both instructive and permissive signaling mechanisms in a functionally redundant manner insulates PLM branching pattern from perturbation in any individual signaling axis.

### VANG-1 promotes Wnt signaling by facilitating Frizzled endocytosis

Endocytosis is a key step in the transduction of Wnt signals in planar cell polarization, growth cone guidance and synaptogenesis [34–38]. We found that the PCP protein VANG-1 is both necessary and sufficient to promote MIG-1 internalization, which extends previous studies of Frizzled endocytosis in neuronal development [25, 37, 39, 40]. A previous study reported that in the commissural axons of the mouse spinal cord, Vangl2 promoted Frizzled3 localization to the intracellular vesicles by antagonizing Dvl1-dependent Frizzled phosphorylation [36]. By contrast, we show that MIG-1 and VANG-1 were all necessary for proper PLM branching, suggesting that they act in the same direction. As the C-terminus of MIG-1 was dispensable for VANG-1 binding, we hypothesize that VANG-1 interact with MIG-1 through other domains.

### Orthogonal signaling cues pattern neurite branching in the two-dimensional space

Development of neurite branches in the 2D or 3D space requires sophisticated coordination between signaling cues distributed along distinct body axes. Our findings that Wnts and Netrin independently specify the position and trajectory of the PLM branch along A-P and D-V axes, respectively, illustrate how orthogonal cues intersect to generate precise neurite branching patterns. While Wnt signaling inhibits and the Netrin-DCC signaling promotes F-actin assembly, both pathways leverage F-actin assembly to control PLM branch development. UNC-40/DCC had been previously shown to directly interact with CED-10/Rac [41], and our data indicate that genetically Wnt-PCP signaling inhibits CED-10/Rac and MIG-2/Rac. It is tempting to speculate that the Rac and Rho small GTPases serve as a shared regulatory step at which signals from orthogonal patterning cues converge to shape neurite branching patterns. An important but unanswered question is how endosomal signaling generates asymmetry in F-actin assembly, the elucidation of which will shed light on the mechanisms that translate directional signaling cues into polarized cytoskeletal activity and compartmentalized morphogenesis patterns of the neurons.

## Materials and methods

### *C*. *elegans* strains and genetics

Strains were cultured and maintained as described [42]. Strains and transgenes used in this study are: LG I: *unc-54(e190)* [43], *mig-1(e1787)* [19], *unc-40(n324)*; LG II: *mig-14(ga62)* [20], *cwn-1(ok546)* [44], *cam-1(gm122)* [45], *dsh-2(ok2162)/mIn1*, *dsh-1(ok1445)*, *mig-5(rh147)* [46]; LG IV: *egl-20(n585)* [47], *cwn-2(ok895)* [44], *prkl-1(ok3182)*, *rabs-5(ok1513)*, *ced-10(n1993)* [48]; LG V: *fmi-1(hd121)* [49], *mom-2(ok591)/nT1*; LG X: *vang-1(tm1422)* [50], *lin-18(e620)* [51], *bar-1(ga80)* [52], *arr-1(ok401)*, *mig-2(mu28)*, *mig-2(gm103gf)* [53], *unc-6(ev400) [54]*, *zdIs5(Pmec-4*::*GFP)*, *muIs42(Pmec-7*::*GFP)*, *jsIs973(Pmec-7*::*RFP)*, *twnEx110(Pmec-7*::*COR-1*::*GFP*, *Pttx-3*::*GFP)*, *twnEx195(Pmec-7*::*COR-1*::*mCherry*, *Pgcy-8*::*mCherry)*, *twnEx202(Pmec-7*::***Δ****CRD*::*MIG-1*::*GFP*, *Pdpy-30*::*NLS*::*dsRed)*, *twnEx205(Phsp*::*CWN-1*, *Pttx-3*::*GFP)*, *twnEx209(Pmec-7*::*MIG-1*::*GFP*, *Pdpy-30*::*NLS*::*dsRed)*, *twnEx230(Pmec-7*::*GFP*::*VANG-1*, *Pdpy-30*::*NLS*::*dsRed)*, *twnEx231(Pmec-7*::*MIG-1*::*GFP*, *Pmec-7*::*myr*::*mCherry*, *Pgcy-8*::*mCherry)*, *twnEx232(Pmec-7*::*GFP*::*VANG-1*, *Pmec-7*::*myr*::*mCherry*, *Pgcy-8*::*mCherry)*, *twnEx254(Pmec-7*::*MIG-1****Δ****C*::*GFP*, *Pmec-7*::*myr*::*mCherry*, *Pgcy-8*::*mCherry)*, *twnEx256 (Pmec-7*::*VAB-10B-ABD*, *Pttx-3*::*GFP)*, *twnEx262(Pegl-20*::*CWN-1*::*Venus*, *Pttx-3*::*GFP)*, *twnEx263(Punc-129*::*UNC-6*, *Pdpy-30*::*NLS*::*dsRed)*, *twnEx266(Pegl-20*::*EGL-20*::*Venus*, *Pttx-3*::*GFP)*, *twnEx265(Phsp*::*EGL-20*, *Pttx-3*::*GFP)*, *twnEx267(Pcwn-2*::*CWN-1*::*Venus*, *Pgcy-8*::*mCherry)*, *twnEx268(Pcwn-2*::*EGL-20*::*Venus*, *Pgcy-8*::*mCherry)*, *twnEx269(Pcwn-2*::*EGL-20*, *Pcwn-2*::*CWN-1*, *Pgcy-8*::*mCherry)*, *twnEx272(Pmec-7*::*MIG-1****Δ****C*::*GFP*, *Pdpy-30*::*NLS*::*dsRed)*, *twnEx273(Pmec-7*::*GFP*::*VANG-1*, *Pmec-3*::*MIG-1*::*mCherry*, *Pttx-3*::*GFP)*, *twnEx275(Pmec-3*::*MIG-1*::*mCherry*, *Pmec-7*::*GFP*::*RAB-5*, *Pttx-3*::*GFP)*, *twnEx276(Pmec-7*::*ARR-1*::*mCherry*, *Pdpy-30*::*NLS*::*dsRed)*, *twnEx337(Pmec-7*::*RHO-1(G14V)*, *Pdpy-30*::*NLS*::*dsRed)*, *twnEx351(Prig-3*::*mCherry*, *Prig-3*::*CD4*::*GFP(1–10)*, *Pmec-7*::*mCherry*, *Pmec-7*::*CD4*::*GFP(11)*, *Pgcy-8*::*GFP)*, *twnEx378(Pmec-7*::*GFP*::*VANG-1*, *Pmec-7*::*COR-1*::*mCherry*, *Pgcy-8*::*mCherry)*, *twnEx379(Pmec-3*::*MIG-1*::*GFP*, *Pmec-7*::*COR-1*::*mCherry*, *Pgcy-8*::*mCherry)*. The transgene *twnEx351* was used in the GRASP experiment (S5 Fig). For detailed information regarding the strains and transgenes used in individual Figures, please see S1 Table. For rescue experiments with transgenes, we examined at least two independent lines of transgenic animals to confirm that the results were reproducible and consistent between these lines. We then used the one that was easier in manipulation and maintenance (higher transmission rate in most cases) for subsequent data acquisition and analyses.

### Measurements and quantification of PLM branch locations

Animals with *zdIs5(Pmec-4*::*GFP)* or *jsIs973(Pmec-7*::*RFP)* were anesthetized by 1% sodium azide, and the PLMs were imaged under the 10X objective of an AxioImager M2 system (Carl Zeiss). The length of the PLM process and the distance between the PLM branch and the cell soma were analyzed using ImageJ [55]. Each animal was only scored for left or right PLM based on which side is clearly visible in the image. There was no side-to-side difference in PLM branch positions, so data from left and right PLMs were pooled for analyses. We define normal PLM branch locations as those that fall within 99% (mean ± 3 standard deviations) of PLM branch sites visualized by *zdIs5(Pmec-4*::*GFP)* in the wild type, which ranges from 0.46 to 0.88. The percentage of both proximally and distally mislocalized PLM branches in indicated genotypes was calculated and presented along with the scatter plots.

### Molecular biology and plasmid construction

The molecular cloning and plasmid construction were performed by standard molecular biology techniques. All of expression constructs used to generate the *twnEx* series of transgenes were in the pPD95.77 Fire vector backbone, including transgenes induced by heat shock (through the *hsp-16*.*2* promoter). Site-direct mutagenesis was performed with QuickChange kit. Detailed information, including primer sequences, for cloning *cor-1*, *vab-10b-ABD*, *cwn-1*, *egl-20*, *mig-1*, *vang-1*, *rab-5*, and *arr-1* are available upon request.

### Heat shock experiments

For heat shock experiments, animals with heat-shock inducible transgenes were grown on NGM plates at 20°C, transferred to 34°C for 30 minutes at early L1 stage, and recovered at 20°C for another 24 hours before the branch locations were scored.

### Western blotting and co-immunoprecipitation

HEK293 cells were transfected by lipofectamine (Invitrogen) and then lysed in lysis buffer (50 mM Tris, 150 mM NaCl, 2 mM EDTA [pH 8.0], 0.5% sodium deoxylcholate, 10 mM phenylmethylsulfonyl fluoride, and 1M dithiothreitol) with 1% NP-40. For co-immunoprecipitation, cell lysates were immunoprecipitated by anti-HA (Invitrogen) or anti-FLAG (Sigma) beads. Immuno-complexes or samples for western blot analysis were electrophoresed in a SDS-polyacryalamide gel, transferred onto PVDF membrane and probed with anti-HA Y-11(1:4000, Santa Cruz) or anti-FLAG (1:5000, Sigma) antibodies.

### Time lapse imaging of PLM branch development

To immobilize worms without interfering with animal development, we used the *unc-54(e190)* mutation to genetically paralyze the animals. The *unc-54* mutation did not affect PLM development. We placed early L1 larva and 2–4 μl polystyrene beads (0.1 μm, Polysciences Inc.) on 5% gel pad. The cover slip was sealed by vaseline to prevent desiccation.

### Confocal microscopy and quantification of MIG-1 and VANG-1 subcellular localization

z-stack maximum projection images were acquired using the Zeiss LSM 700 Confocal Imaging System (Carl Zeiss). Pixel-wise colocalization of GFP and mCherry fluorescence signal was quantified using the Zeiss Zen imaging software. For the quantification of MIG-1 or VANG-1 cytosolic localization, myristolated mCherry was expressed to label the cell membrane of the touch neurons. Using Zeiss Zen software, total and cytosolic MIG-1::GFP signal intensity was derived by first quantifying each single optical sections of the z-stack confocal images that span most of the thickness of the PLM cell body, followed by summation of data from individual optical sections to calculate the cytosolic/total signal intensity ratio.

### Analysis of COR-1 localization

Heat maps of COR-1::mCherry intensity in the PLM process were generated from z-stack maximum projection images using ImageJ. Ten individual PLMs were assembled into a single aligned heat map of COR-1 intensity for each genotype.

### Statistics analysis

The ANOVA, Student’s *t* test, Mann-Whitney U test and two proportion z test were conducted in MS Office Excel or Prism for experiments indicated in the Figure Legends, with Bonferroni correction for multiple comparisons. Error bars represent standard error of means (S.E.M.).

### Ethics statement

This study does not involve any human subject, non-human primates and other vertebrates.

## Supporting information

S1 Fig The PLM branching pattern during larval development. (A, B) Quantification of (A) PLM branching sites or (B) the number of PLM branch at different developmental stages. Synchronized animals were analyzed at indicated developmental stages. N > 30.
(TIF)

S2 Fig Neurite growth and F-actin distribution in the Wnt mutants. (A) The length of the PLM neurite in the *mig-14*, *cwn-1* or *egl-20* mutants. N > 30. (B) The number of PLM branch in the *cwn-1; egl-20* at different developmental stages. N > 30. (C) Heat maps (N = 10) of COR-1::mCherry*(twnEx195)* intensity in the PLM process of indicated mutants at 7 hours post-hatching.
(TIF)

S3 Fig MIG-1 clustering is dependent on EGL-20 but not CWN-1. (A) Confocal fluorescent images of MIG-1::GFP in the PLM soma of indicated genotypes. Scale bar = 5 μm. (B) Confocal images of MIG-1::GFP in the PLM process. Scale bar = 10 μm. Asterisks, PLM soma. Arrows indicate MIG-1 signals from the PLM on the other side.
(TIF)

S4 Fig The CRD and C-terminal domains are dispensable for MIG-1 and VANG-1 interaction. Co-immunoprecipitation of MIG-1ΔC or MIG-1ΔCRD with VANG-1. HA::VANG-1 or MIG-1 variants tagged with FLAG were expressed in HEK293 cells. Cell lysates were immunoprecipitated by beads coated with Y-11(anti-HA) or M2(anti-FLAG) antibodies, and subsequently analyzed by western blotting. Arrows and arrowheads indicate VANG-1 and MIG-1 variant bands, respectively.
(TIF)

S5 Fig Synaptic contact is not affected in the *mig-1* mutant. GRASP (GFP reconstitution across synaptic partners) signal in the wild type and the *mig-1* mutants. Two GFP fragments, GFP(11) and GFP(1–10), were fused to the transmembrane protein CD4 and expressed in the touch neurons and the interneurons by the *mec-7* and the *rig-3* promoter, respectively. These promoters are also used to express soluble mCherry to mark the neurites of the PLM and interneurons between which chemical synapses form. In the wild type, reconstituted GFP fluorescence was observed where the presynaptic varicosity of PLM contacted the processes of interneurons. GRASP signal in the *mig-1* mutant was indistinguishable from that of the wild-type animal, indicating that the misplaced PLM branch still formed synaptic contact with postsynaptic interneurons. Scale bar = 5 μm.
(TIF)

S1 Table List of strains used in figures. (DOCX)
