# Supplementary figures and images for: A Wnt-planar polarity pathway instructs neurite branching by restricting F-actin assembly through endosomal signaling
Source: PLoS Genet. 2017 Apr 6;13(4):e1006720. doi: 10.1371/journal.pgen.1006720 (PMC5398721; doi:10.1371/journal.pgen.1006720)

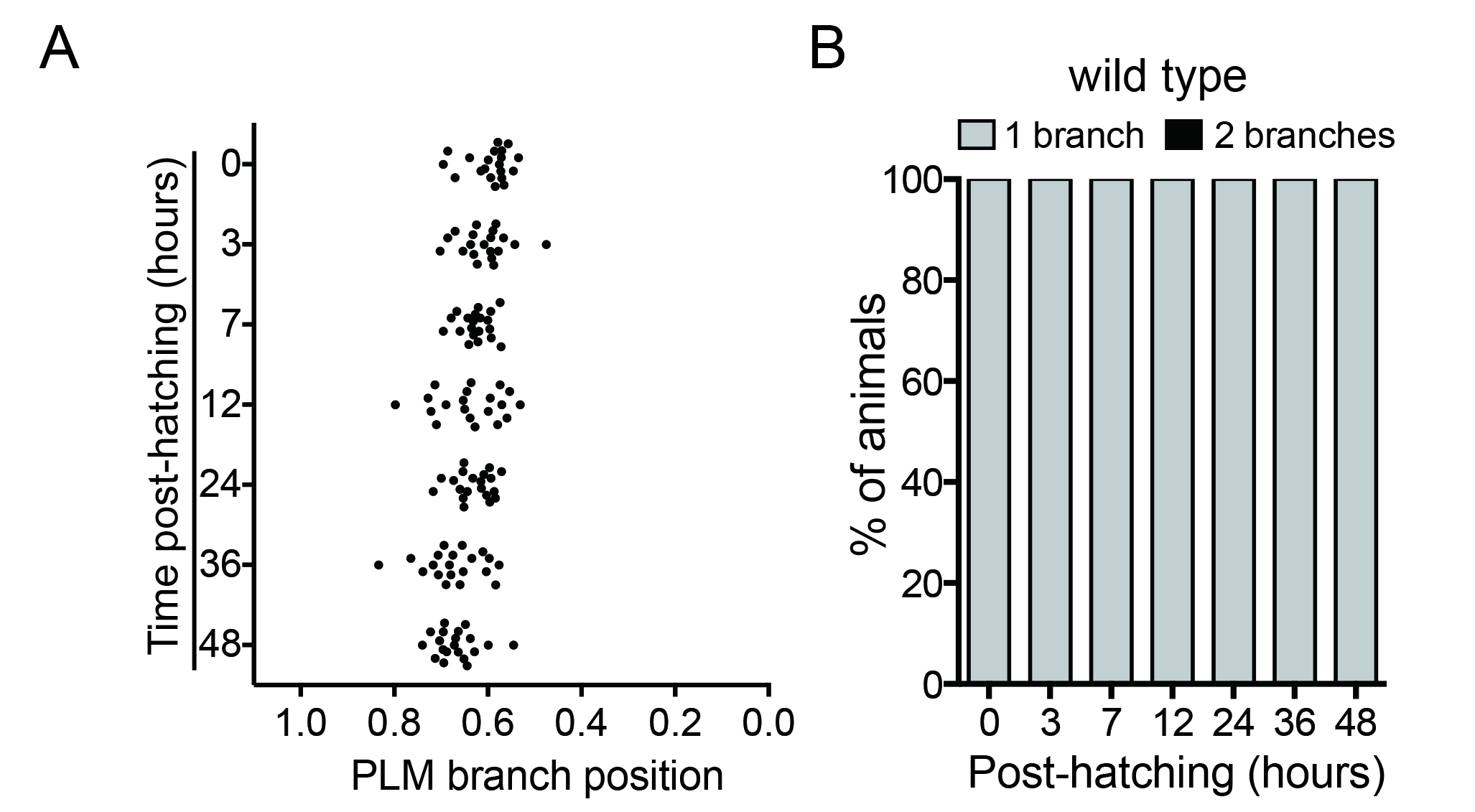

Supplement: S1 Fig — (A, B) Quantification of (A) PLM branching sites or (B) the number of PLM branch at different developmental stages. Synchronized animals were analyzed at indicated developmental stages. N > 30. (TIF) [file pgen.1006720.s001.tif]

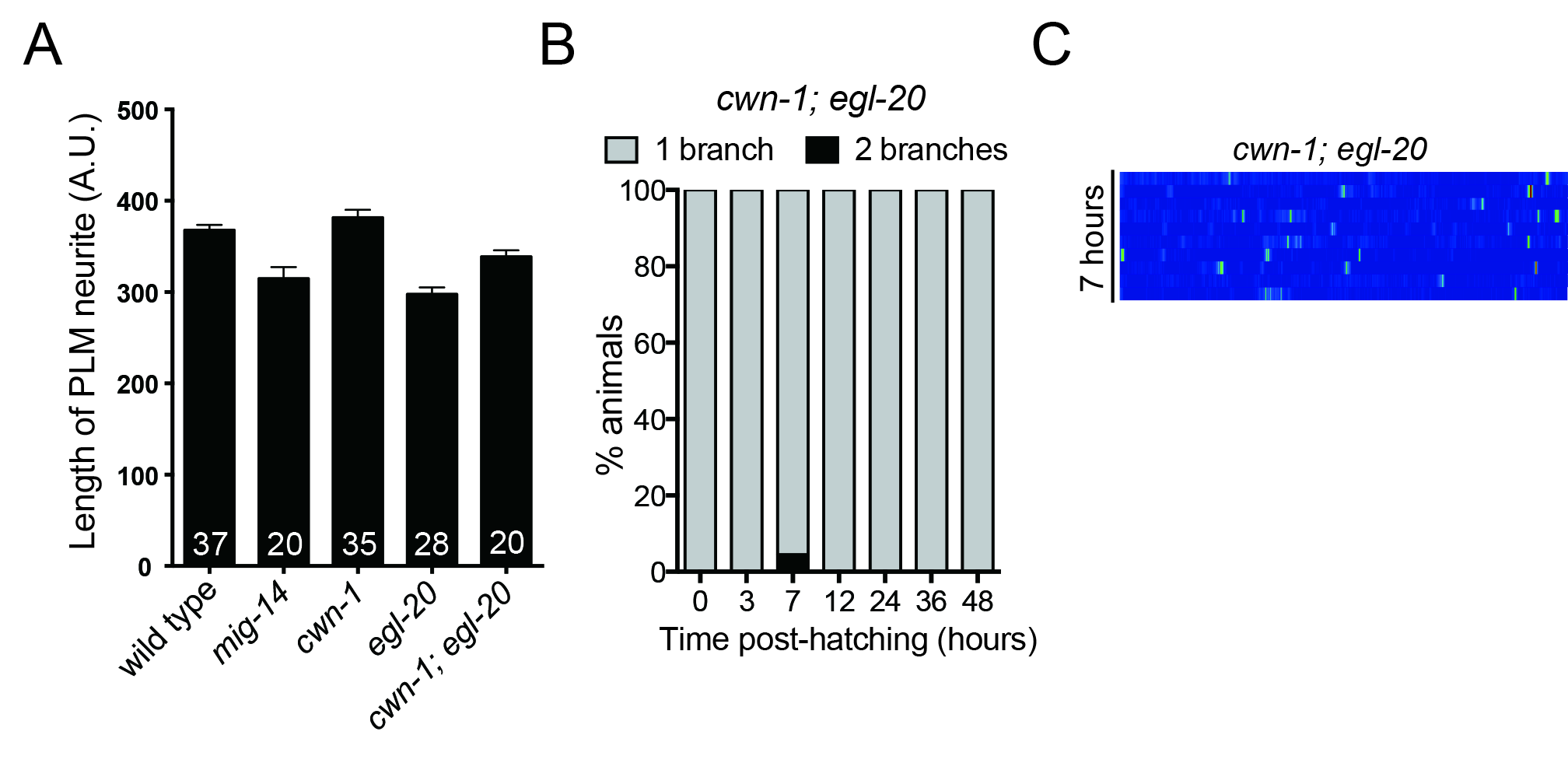

Supplement: S2 Fig — (A) The length of the PLM neurite in the mig-14, cwn-1 or egl-20 mutants. N > 30. (B) The number of PLM branch in the cwn-1; egl-20 at different developmental stages. N > 30. (C) Heat maps (N = 10) of COR-1::mCherry(twnEx195) intensity in the PLM process of indicated mutants at 7 hours post-hatching. (TIF) [file pgen.1006720.s002.tif]

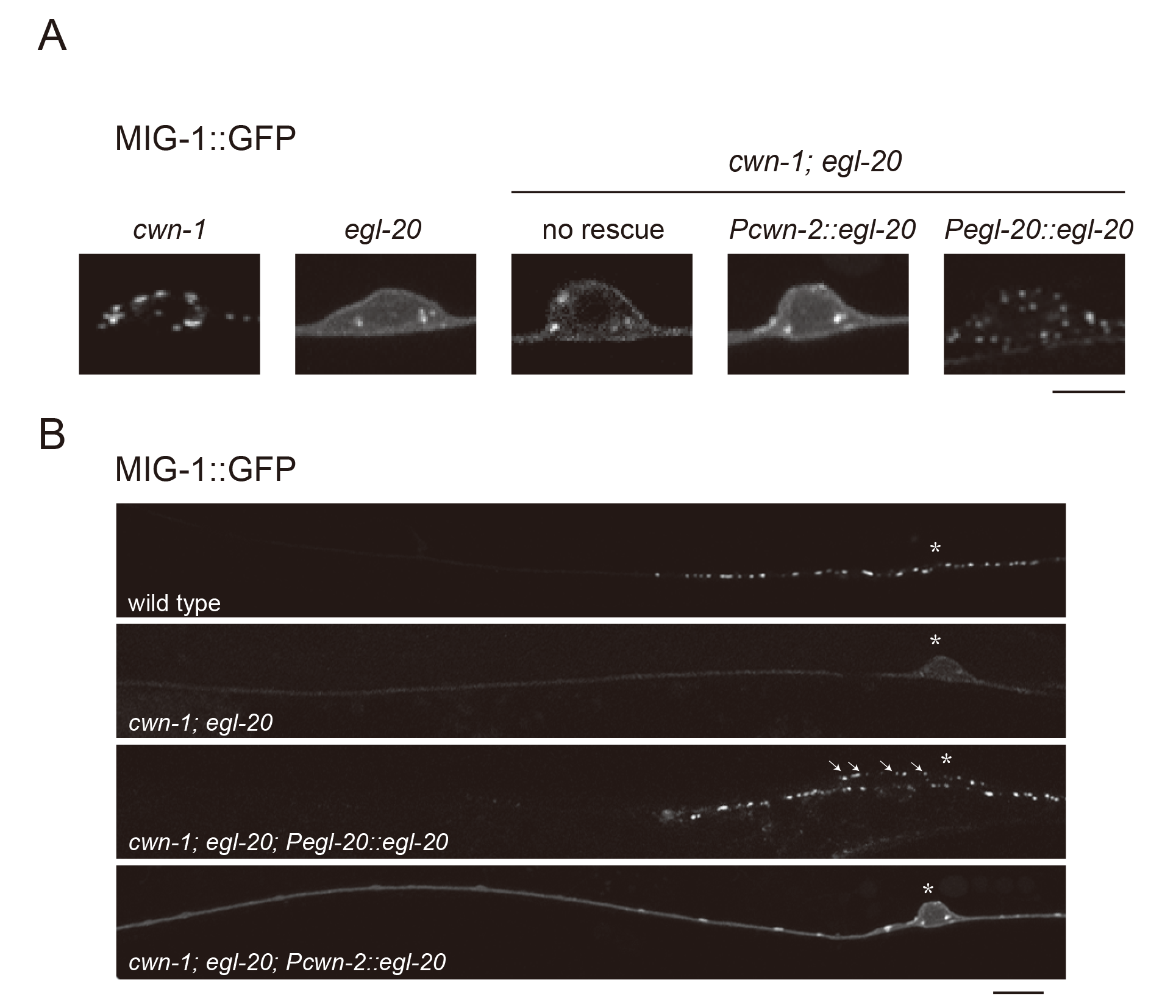

Supplement: S3 Fig — (A) Confocal fluorescent images of MIG-1::GFP in the PLM soma of indicated genotypes. Scale bar = 5 μm. (B) Confocal images of MIG-1::GFP in the PLM process. Scale bar = 10 μm. Asterisks, PLM soma. Arrows indicate MIG-1 signals from the PLM on the other side. (TIF) [file pgen.1006720.s003.tif]

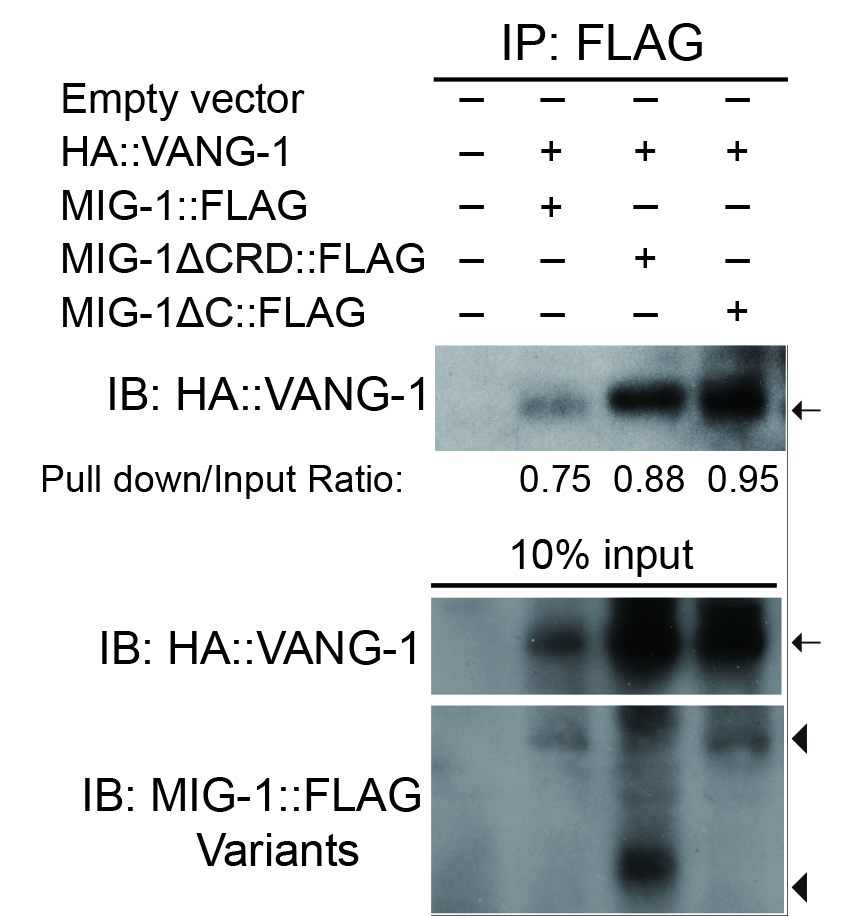

Supplement: S4 Fig — Co-immunoprecipitation of MIG-1ΔC or MIG-1ΔCRD with VANG-1. HA::VANG-1 or MIG-1 variants tagged with FLAG were expressed in HEK293 cells. Cell lysates were immunoprecipitated by beads coated with Y-11(anti-HA) or M2(anti-FLAG) antibodies, and subsequently analyzed by western blotting. Arrows and arrowheads indicate VANG-1 and MIG-1 variant bands, respectively. (TIF) [file pgen.1006720.s004.tif]

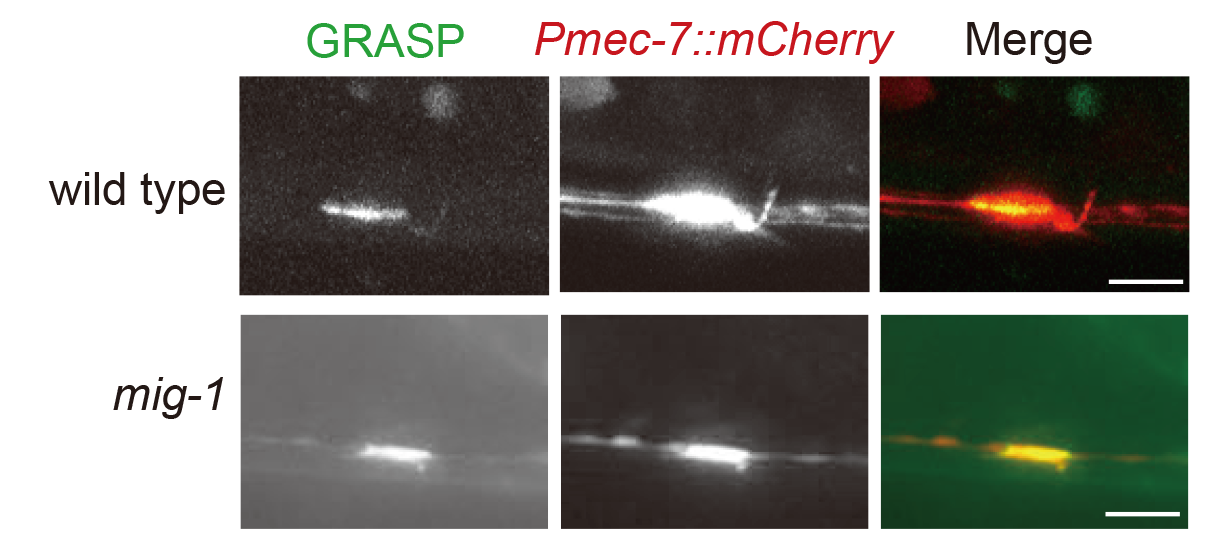

Supplement: S5 Fig — GRASP (GFP reconstitution across synaptic partners) signal in the wild type and the mig-1 mutants. Two GFP fragments, GFP(11) and GFP(1–10), were fused to the transmembrane protein CD4 and expressed in the touch neurons and the interneurons by the mec-7 and the rig-3 promoter, respectively. These promoters are also used to express soluble mCherry to mark the neurites of the PLM and interneurons between which chemical synapses form. In the wild type, reconstituted GFP fluorescence was observed where the presynaptic varicosity of PLM contacted the processes of interneurons. GRASP signal in the mig-1 mutant was indistinguishable from that of the wild-type animal, indicating that the misplaced PLM branch still formed synaptic contact with postsynaptic interneurons. Scale bar = 5 μm. (TIF) [file pgen.1006720.s005.tif]
