# Supplementary material for: A Wnt-planar polarity pathway instructs neurite branching by restricting F-actin assembly through endosomal signaling
Source: PLoS Genet. 2017 Apr 6;13(4):e1006720. doi: 10.1371/journal.pgen.1006720 (PMC5398721; doi:10.1371/journal.pgen.1006720)
Supplement: S1 Table — (DOCX) [file pgen.1006720.s006.docx]

**S1 Table. List of Strains used in Figures**

| Strain name/genotype | Figure |
| --- | --- |
| N2 | 1 |
| CF702 *muIs32[Pmec-7::gfp]* | 1 |
| *jsIs973[Pmec-7::mRFP]* | 1 |
| SK1006 *zdIs5[Pmec-4::GFP, lin-15(+)]* | 1-7 |
| CLP572 *twnEx195[Pmec-7::COR-1::mCherry, Pgcy-8::mCherry]* | 2, 3, 4, 6, 7, S2 |
| CLP418 *twnEx110[Pmec-7::COR-1::GFP, Pttx-3::gfp]* | 2 (E, F) |
| CLP715 *twnEx256[Pmec-7::VAB-10B-ABD::mCherry, Pgcy-8::mCherry]* | 2 |
| CLP724 *unc-54(e190); jsIs973; twnEx110* | 2 (E, F) |
| NG5177 *zdIs5; mig-14(ga62)* | 3 |
| *zdIs5; cwn-1(ok546)* | 3, S3 |
| *zdIs5; cwn-2(ok895)* | 3 |
| NG5778 *zdIs5; egl-20(n585)* | 3, S3 |
| NG4616 *zdIs5; cwn-1(ok546); egl-20(n585)* | 3, S2, S3 |
| CLP582 *zdIs5; cwn-1(ok546); egl-20(n585); twnEx195* | 3 |
| CLP438 *zdIs5; cwn-1(ok546); cwn-2(ok895) egl-20(n585)* | 3 |
| CLP609 *zdIs5; twnEx205[Phsp16.2::CWN-1, Pttx-3::GFP]* | 3 |
| *zdIs5; twnEx265[Phsp16.2::EGL-20, Pttx-3::GFP]* | 3 |
| CLP718 *twnEx262[Pegl-20::CWN-1::Venus, Pttx-3::GFP]* | 3 |
| *zdIs5; cwn-1; egl-20; twnEx267[Pcwn-2::CWN-1::Venus, Pgcy-8::mCherry]* | 3 |
| CLP610 *zdIs5; cwn-1; twnEx114[Pcwn-2::GFP, Pcwn-2::CWN-1]* | 3 |
| *zdIs5; cwn-1; egl-20; twnEx268[Pcwn-2::EGL-20::Venus, Pgcy-8::mCherry]* | 3, S3 |
| *zdIs5; cwn-1; egl-20; twnEx266[Pegl-20::EGL-20::Venus, Pttx-3::GFP]* | 3, S3 |
| CLP384 *zdIs5 mig-1(e1787)* | 4, 5, 6, 7 |
| CLP439 *zdIs5; cam-1(gm122)* | 4 |
| CLP391 *zdIs5; dsh-2(ok2162)/mIn1* | 4 |
| CLP441 *zdIs5; dsh-1(ok1445)* | 4 |
| CLP711 *zdIs5; mig-5(rh147)/mIn1* | 4 |
| CLP386 *zdIs5; prkl-1(ok3182)* | 4 |
| *zdIs5; fmi-1(hd121)* | 4 |
| *zdIs5; mom-2(ok591)/nT1* | 4 |
| NG4978 *zdIs5; vang-1(tm1422)* | 4 |
| CLP388 *zdIs5; lin-18(e620)* | 4 |
| CLP607 *zdIs5; bar-1(ga80)* | 4 |
| CLP165 *zdIs5; mig-2(mu28)* | 4 |
| CLP62 *zdIs5; mig-2(gm103gf)* | 4 |
| CLP161 *zdIs5; ced-10(n1993)* | 4 |
| CLP612 *zdIs5 mig-1; twnEx209(Pmec-7::MIG-1::GFP, Pdpy-30::NLS::dsRed),* | 4 |
| CLP736 *zdIs5 vang-1; twnEx230(Pmec-7::GFP::VANG-1, Pdpy-30::NLS::dsRed)* | 4 |
| CLP576 *zdIs5 mig-1; twnEx195* | 4 |
| CLP573 *zdIs5; vang-1; twnEx195* | 4 |
| *zdIs5 mig-1; twnEx379[Pmec-3::MIG-1::GFP, Pmec-7::COR-1::mCherry, Pgcy-8::mCherry]* | 4 |
| *zdIs5; vang-1; twnEx378[Pmec-7::GFP::VANG-1, Pmec-7::COR-1::mCherry, Pgcy-8::mCherry]* | 4 |
| CLP862 *zdIs5; twnEx337(Pmec-7::RHO-1(G14V), Pdpy-30::NLS::dsRed)* | 4 |
| *twnEx231(Pmec-3::MIG-1::GFP, Pmec-7::myr::mCherry, Pgcy-8::mCherry)* | 5 |
| CLP661 *twnEx232(Pmec-7::GFP::VANG-1, Pmec-7::myr::mCherry, Pgcy-8::mCherry),* | 5 |
| *twnEx273(Pmec-7::GFP::VANG-1, Pmec-3::MIG-1::mCherry, Pttx-3::GFP)* | 5 |
| *zdIs5 mig-1; twnEx272(Pmec-7::MIG-1****Δ****C::GFP, Pdpy-30::NLS::dsRed),* | 5 |
| CLP732 *zdIs5 mig-1; twnEx202(Pmec-7::****Δ****CRD::MIG-1::GFP, Pdpy-30::NLS::dsRed)* | 5 |
| *twnEx275(Pmec-3::MIG-1::mCherry, Pmec-7::GFP::RAB-5, Pttx-3::GFP)* | 6 |
| *vang-1; twnEx231(Pmec-3::MIG-1::GFP, Pmec-7::myr::mCherry, Pgcy-8::mCherry)* | 6 |
| *arr-1; twnEx231(Pmec-3::MIG-1::GFP, Pmec-7::myr::mCherry, Pgcy-8::mCherry)* | 6 |
| CLP713 *twnEx254(Pmec-7::MIG-1****Δ****C::GFP, Pmec-7::myr::mCherry, Pgcy-8::mCherry)* | 6 |
| NG6177 *zdIs5; rabs-5(ok1513)* | 6 |
| NG6175 *zdIs5; arr-1(ok401)* | 6 |
| NG6407 *zdIs5 mig-1; arr-1(ok401)* | 6 |
| CLP747 *twnEx276(Pmec-7::ARR-1::mCherry, Pdpy-30::NLS::dsRed)* | 6 |
| *zdIs5; unc-6(ev400)* | 7 |
| CLP126 *zdIs5 unc-40(n324)* | 7 |
| *zdIs5 unc-40(n324); Pmec-7::UNC-40* | 7 |
| CLP645 *zdIs5 mig-1; unc-6* | 7 |
| CLP719 *zdIs5; twnEx263(Punc-129::UNC-6, Pdpy-30::NLS::dsRed)* | 7 |
| CLP745 *zdIs5 mig-1; twnEx263(Punc-129::UNC-6, Pdpy-30::NLS::dsRed)* | 7 |
| *twnEx351(Prig-3::mCherry, Prig-3::CD4::GFP(1-10), Pmec-7::mCherry, Pmec-7::CD4::GFP(11), Pgcy-8::GFP)* | S5 |
